# Modelling sexual violence in male rats: the sexual aggression test (SxAT)

**DOI:** 10.1038/s41398-022-01973-3

**Published:** 2022-05-18

**Authors:** Vinícius E. de M. Oliveira, Trynke R. de Jong, Inga D. Neumann

**Affiliations:** 1 Department of Behavioural and Molecular Neurobiology, University of Regensburg, 93040 Regensburg, Germany; 2 Laboratory of Neuroendocrinology, GIGA-Neurosciences, University of Liege, 4020 Liege, Belgium; 3 Medische Biobank Noord-Nederland B.V, 9713 Groningen, Netherlands

**Keywords:** Neuroscience, Pharmacology, Psychiatric disorders

## Abstract

Sexual assault and rape are crimes that impact victims worldwide. Although the psychosocial and eco-evolutionary factors associated with this antisocial behavior have repeatedly been studied, the underlying neurobiological mechanisms are still largely unknown. Here, we established a novel paradigm to provoke and subsequently assess sexual aggression (SxA) in adult male Wistar rats: the sexual aggression test (SxAT). Briefly, male Wistar rats are sexually aroused by a receptive female, which is exchanged by a non-receptive female immediately after the first intromission. This protocol elicits forced mounting and aggressive behavior toward the non-receptive female to different degrees, which can be scored. In a series of experiments we have shown that SxA behavior is a relatively stable trait in rats and correlates positively with sexual motivation. Rats with innate abnormal anxiety and aggressive behavior also show abnormal SxA behavior. In addition, central infusion of oxytocin moderately inhibits aggressive behavior, but increases forced mounting. Finally, we identified the agranular insular cortex to be specifically activated by SxA, however, inhibition of this region did not significantly alter behavior in the SxAT. Altogether, the SxAT is a paradigm that can be readily implemented in behavioral laboratories as a valuable tool to find answers regarding the biological mechanisms underlying SxA in humans, as well as social decision-making in general.

## Introduction

According to the World Health Organization (WHO), worldwide, about 35% of women have experienced physical and/or sexual violence in their lifetime [1], which is even likely to be an underestimation [2]. Rape and sexual assault have severe physical and mental consequences including post-traumatic stress disorder [3, 4]. Thus, effective prevention of sexual violence is highly warranted, but depends largely on the identification of factors that facilitate or inhibit a person’s tendency to repeatedly assault or rape. Unfortunately, current risk assessments and therapies aimed to prevent recidivism of sex offenders lack adequate efficacy [5–7].

Traditionally, the motivating factors contributing to (male-to-female) sexual aggression (SxA) have been studied in two relatively separated scientific worlds. On the one hand, ecologists and evolutionary biologists have shown that sexual conflict is common in many animal species, and driven by the evolutionary forces of reproductive success and failure [8–11]. On the other hand, sociologists, psychologists, and psychiatrists have used questionnaires and diagnostic instruments to formulate theoretical frameworks modeling the important roles of culture, childhood experiences, and personality traits in the human tendency to rape [12, 13].

While it is certainly important to understand the evolution of SxA throughout the animal kingdom and rape culture in humans, empirical data on genetic, hormonal, and neuroanatomical factors underlying an individual’s tendency to display SxA are essential in order to seek effective therapies. Unfortunately, there are only anecdotal descriptions of biological factors identified in (violent) sex offenders. Thus, white matter abnormalities in cortical and subcortical brain areas associated with moral decision-making, reward-processing, sexual arousal, and aggression [14, 15], and increased testosterone or gonadotrophic hormones levels moderately predicting recidivism and hostility, have been described [16, 17].

Detailed investigations into the neurobiology of SxA can only be promoted using a validated animal model of SxA under controlled laboratory conditions. Especially rodent models with relatively homogeneous sexual, aggressive, and sexually aggressive behaviors enable a wide variety of experimental interventions to be tested. Rodent models have been successfully used for decades to investigate the neuronal mechanisms of various (dysfunctional) social behaviors including social decision-making, empathy, consolation, social reciprocity, social fear and social avoidance, maternal behavior, and male or female aggression [reviewed in [18, 19]]. Aggression in a sexual context is a notable exception in this list.

Therefore, our main aim was to establish a robust test to assess SxA in male rats that could be easily implemented in other behavioral laboratories. Appreciating the enormous complexity and variety of contexts of SxA in humans (i.e., rape and assault), we narrowed our aim down to focus on the most basic and translational aspects of SxA, i.e,. a sexually aroused male persisting in forced sexual acts and showing aggressive behavior toward a non-willing and defensive female.

In the process of establishing the first rat model of SxA, we first assessed the behavioral variability in sexually aroused male Wistar rats presented with a non-receptive female, i.e., scoring their persistence in mounting the non-willing female combined with aggression toward that female. Secondly, as personality traits [12, 15] have been linked with sexual violence, we investigated, whether innate differences in anxiety and social behaviors are associated with the display of SxA in rats selectively bred for high (HAB) vs. low (LAB) anxiety-related behavior [20, 21]. For further validation of the SxA model, we tested the effects of pharmacological treatments hypothesized to affect SxA. We chose synthetic oxytocin (OXT) and arginine vasopressin (AVP), both known to affect pro-social interactions, aggression and sexual behavior in male rats [18, 19, 22–33], and ethanol known to play a role in human rape and assault [29] and in the (dis)inhibition of aggressive and sexual behavior in rodents [34–36]. We then analyzed the patterns of neuronal activity in response to either SxA, consensual mating, or territorial aggression in brain areas selected for their known involvement in aggression, consensual mating, and/or social decision-making [37–41]. Finally, we measured the effects of functional inhibition of the brain area in which the activation pattern most strongly correlated to SxA: the posterior agranular insular cortex (pAIC), which is also known to be strongly involved in social-decision making, empathy, sexual and aggressive behaviors [15, 42, 43].

## Materials & methods

### Ethics statement

Experiments were approved by the Committee on Animal Health and Care of the Government of the Unterfranken (2532-2-443) and followed the Guide for the Care and Use of Laboratory Animals produced by the National Institute of Health. Sample size was determined based on the magnitude and consistency between groups and/or experimental conditions as well as in previous experiments.

### Animals

Experiments were carried out in adult (12–15 weeks) male Wistar rats, which were bred in the animal facilities of the University of Regensburg, Germany, and either non-selected (NAB), or selectively bred for high (HAB) or low (LAB) anxiety-related behavior [20, 21]. As stimulus animals during the various behavioral tests, young (10–12 weeks) adult female or male NAB Wistar rats, either bred at the animal facilities or obtained from Charles Rivers Laboratories (Sulzfeld, Germany), were used. All females were intact and naturally cycling. All rats were kept under controlled laboratory conditions (12:12 h light/dark cycle; lights off at 11:00 h, 21 ± 1 **°**C, 60 ± 5% humidity, standard rat nutrition (RM/H, Ssniff Spezialdiäten GmbH, Soest, Germany) and water *ad libitum*) and housed in groups of four in standard rat cages (55 × 35 × 20 cm) with sawdust bedding until the start of the experimental procedures. All behavioral tests were performed in the first half of the dark phase, between 12:00 h and 17:00 h. In addition, 48 h prior to the behavioral procedures (except the elevated plus-maze (EPM)) the experimental males were single-housed in experimental observation cages (home cage, 40 × 24 × 35 cm, plexiglas walls).

### Behavioral tests

#### Sexual aggression test (SxAT)

We established the SxAT to initially provoke SxA and to assess the individual degree of SxA. To perform the SxAT, the (non-)receptivity of the required stimulus females was screened in the morning by observing the presence or absence of hopping, darting, and lordosis during a brief encounter with a non-experimental sexually experienced male Wistar rat.

The SxAT consisted of two distinct, consecutively performed parts: an arousal phase and an agonistic phase. In the first phase, sexual arousal was achieved by introducing a receptive female into the male’s home cage; the latency to mount the receptive female (with or without intromission) was measured as copulation latency. As soon as the male performed either 3 mounts or 1 intromission (whatever occurred first), or until 5 min elapsed without any mount or intromission, the receptive female was replaced by a non-receptive female, thus starting the agonistic phase. During the following 10 min, all signs of SxA, i.e., the frequency of forced mounts and aggressive behaviors by the male were scored (Supplementary video 1).

Forced mounts were counted, when the male clearly initiated a mount (i.e., accelerating its pursuit of the female, attempting to place its forepaws on the female’s flanks, thrusting its pelvis forward), which was clearly rejected by the female [40]. We distinguished four rejection strategies by the female, often accompanied by audible distress vocalizations: (i) passively keeping a non-lordotic posture, (ii) kicking back with hind paws, (iii) turning around upright to face the male (sometimes accompanied by boxing), or (iv) lying down on the back. Forced mounts often, but not always, resulted in the males’ pelvis touching some part of the female body (depending on the rejection strategy of the female), but never resulted in a vaginal intromission. In ~5% of SxATs, the previously non-receptive female started to show receptive behavior. In that case, the female was immediately removed from the cage and replaced by another, non-receptive, stimulus female.

Aggressive behavior comprised of all behaviors typically displayed by rats during territorial conflicts and scored in the resident-intruder test (RIT) [44, 45]: “forced grooming” (male aggressively licking the head/neck area of the female), “keep down” (male using his front paws and upper body to force the female to lie on her back), “threat” (male displaying a threatening posture or movement toward the female, including pushing/shoving the female with his head), “lateral threat” (male turning his body sideways and pushing the female into a wall or corner) and “attack” (rapid clinch attack with one or more bites). Females regularly displayed the same types of aggressive behavior toward the males, but to a much lesser extent.

The copulation latency, and the occurrence of forced mounting and aggressive behavior were first scored live by a trained observer using a stopwatch and a standardized pen-and-paper score form. For forced mounts and aggressive behaviors, this resulted in a frequency score (number of behaviors in 10 min). All SxATs were also recorded with an infrared video camera (Sumikon, PEARL GmbH, Buggingen, Germany). For pharmacological experiments (Exp 3 and Exp 5), the full spectrum of behaviors throughout the SxAT was scored from video using JWatcher behavioral observation software [46] in order to detect any changes due to the treatments. Thus, aside from forced mounting and aggressive behavior, also neutral behaviors (immobility, exploration, eating/drinking, auto grooming) and non-aggressive social interactions (anogenital sniffing, defensive behavior) were scored. From these scores, the percentage of time that an animal spent performing a specific type of behavior was calculated (total duration of behavior/10 min × 100%).

#### Consensual mating test (CMT)

To assess ‘normal’ sexual behavior of experimental males, the consensual mating test (CMT) was performed as described [47]. A receptive female was placed in the home cage of the experimental male, i.e., in a non-paced mating condition [48], and free interactions were allowed for 10 min. Mounting, copulation, and ejaculation latencies, as well as respective frequencies, were scored either live or on video recordings using JWatcher software.

#### Resident intruder test (RIT)

To assess intermale territorial aggression, the RIT was performed as described [44]. Briefly, a virgin male intruder, weighing ~20% less than the experimental resident male, was placed in the home cage of the resident for 10 min. Various social and non-social behaviors of the resident also scored during the SxAT (please see Sexual aggression test. for details) were recorded on an infrared video camera and analyzed using JWatcher software.

#### Elevated plus-maze (EPM) test

For analysis of anxiety-related behavior, rats were tested on the EPM [20, 21] as described in detail in the supplementary information.

### Stereotaxic surgery

Stereotaxic surgery was performed as previously described [26, 27], for further information please see supplementary information.

### Pharmacological interventions

All pharmacological treatments were infused by a trained experimenter blind to the treatments.

#### I.c.v. OXT or AVP infusion

Synthetic OXT and AVP (Sigma-Aldrich Biochemicals, Munich, Germany) were dissolved in Ringer’s solution (VEH, pH = 7.4, B. Braun AG, Melsungen, Germany). OXT was infused i.c.v. at a dose of 1 or 100 ng/5 µl [26, 27], and AVP was infused at a dose of 0.1 or 1 ng/5 µl [26] 10–15 min prior to the SxAT.

#### I.p. Ethanol injection

Ethanol (Fortior Primasprit Neutralalkohol, Brüggemann Alkohol, Heilbronn, Germany) was dissolved in saline (VEH) at 15% w/v, and injected i.p. at either 0.5 g/kg or 1.5 g/kg 10 min prior to the SxAT.

#### Infusion of Baclofen and Muscimol into the posterior agranular insular cortex (pAIC)

The GABA-A receptor agonist muscimol (Sigma Aldrich GmbH, Munich, Germany) and the GABA-B receptor agonist baclofen (Sigma Aldrich GmbH, Munich, Germany) were dissolved together in saline at a dose of 20 ng/μl (muscimol) and 200 ng/μl (baclofen), respectively, and 1 μl of this cocktail was slowly infused into the left and right pAIC 5 min before the start of the SxAT.

### Immunohistochemistry and microscopical analyses

Immunohistochemistry was performed as previously described [27]; for further information about analysis, protocol and abbreviations please see supplementary information. Microscopical analyses were performed by trained observers blind to the experimental groups.

### Experimental protocols

#### Behavioral profiling of SxA (Experiment 1)

In this experiment the aim was to assess (i) the general occurrence of SxA, (ii) the individual variability in SxA among male NAB Wistar rats (*n* = 126), (iii) the SxA behavior of the male as a function of the female response, and (iv) the correlations between SxA behaviors and sexual motivation in Wistar rats. Therefore, the copulation latency in the arousal phase and the number of forced mounts and aggressive behaviors in the 10 min agonistic phase were scored live during three SxATs performed on 3 consecutive days, i.e., SxAT-1/2/3 (described in Sexual aggression test). The female responses and aggressive behaviors were scored as well.

Note that some of these rats (*n* = 32) underwent the three SxA tests (SxAT-1/2/3) specifically for experiment 1, whereas the other rats (*n* = 94) were exposed to three consecutive SxATs as a ‘training’ before the pharmacological treatments of experiment 3. Of this latter group, 57 rats had an i.c.v. cannula implanted 3 days prior to SxAT-1 and were handled before each SxAT (see description in Experiment 3).

#### SxA in HAB, LAB, and NAB rats (Experiment 2)

To determine whether an innate high or low level of anxiety-related behavior influences SxA, HAB, LAB, and NAB rats (*n* = 10 each) were tested in three SxAT performed on 3 consecutive days (SxAT-1/2/3). High, low, and normal anxiety-related behavior was confirmed on the EPM (supplementary information) either 1 week before (HAB/LAB) or after (NAB) testing in the SxAT.

The copulation latency in the arousal phase and the number of forced mounts and aggressive behaviors in the agonistic phase were scored live. The standard SxAT paradigm (described in Sexual aggression test) had to be modified for this particular experiment to protect stimulus females against the high levels of aggression, including lateral threats and severe attacks, displayed by the LAB and (to a lesser extent) HAB rats, confirming prior findings [49, 50] Therefore, the SxAT was curtailed to 5 rather than 10 min, and the arousal phase was skipped in SxAT-2 (i.e., only the agonistic phase was performed).

The difference in quality of aggressive behavior between the strains was quantified using the proportion of lateral threats and attacks in the total frequency of aggressive behaviors, abbreviated as LTA% (=[the number attacks and lateral threats]/[the number of all aggressive behaviors] × 100%, with 0/0 counted as 0).

#### Pharmacological manipulation of SxA (Experiment 3)

For pharmacological manipulation, rats fitted with an i.c.v. cannula 3 days prior to SxAT-1 (supplementary information) were first trained in three SxATs performed on 3 consecutive days (SxAT-1/2/3). The behavioral results from these training sessions were added to the analyses in experiment 1. The training phase served the purpose to (a) habituate males to the administration procedure, (b) stabilize SxA, (c) exclude animals with consistently low SxA scores from the experiment (*n* = 4), and (d) equally distribute the remaining animals over the experimental groups to achieve balanced average SxA levels. One rat was removed from the experiment due to lethargic behavior in the home cage.

Effects of i.c.v. pharmacological treatments were assessed in SxAT-4, performed one day after SxAT-3. For experiment 3a, rats were infused with either VEH (*n* = 9), 1 ng/5 µl of OXT (OXT-1, *n* = 10) or 100 ng/5 µl of OXT (OXT-100, *n* = 9) 15 min prior to SxAT-4 (see i.c.v. OXT and AVP infusion).

For experiment 3b, rats were infused with either VEH (*n* = 9), 0.1 ng/5 µl of AVP (AVP-0.1, *n* = 8) or 1 ng of AVP/5 µl (AVP-1, *n* = 10) 10 min prior to SxAT-4 (see i.c.v OXT and AVP infusion).

For experiment 3c, rats were i.p. injected with either saline (VEH, *n* = 11), 0.5 mg/kg ethanol (ETH-0.5, *n* = 11) or 1.5 mg/kg (ETH-1.5, *n* = 12) 10 min prior to SxAT-4 (see i.p. Ethanol injection).

In experiments 3a, b, and c, exposure to the SxAT-4 was followed immediately by a 5 min CM (see Consensual mating test) to assess whether the pharmacological treatments affected general sexual motivation and execution. Animals who responded to treatment with abnormal behavior (for example extensive immobility or excessive auto grooming) were removed from statistical analyses (OXT-1: *n* = 1; OXT-100: *n* = 2; AVP-1: *n* = 1; ETH-1.5: *n* = 4).

To capture the full spectrum of behaviors, all experimental SxAT’s in experiment 3 were scored from video using J-Watcher by trained observers blind to the pharmacological treatment. The percentage of time spent displaying forced mounts, aggressive behaviors, neutral behavior and social behavior in the 10-min tests were calculated.

#### Neuronal activation in response to SxA (Experiment 4)

To monitor the neuronal activity in response to SxA within relevant brain regions, male Wistar rats (*n* = 36) were exposed to three CMTs performed on 3 consecutive days to (a) habituate them to the behavioral test set-up, (b) to gain an equal level of sexual experience, and (c) to facilitate interactions with stimulus animals on the final experimental day 4.

On day 4, males were equally distributed to four experimental groups, receiving either no stimulus (control group), 10 min exposure to a non-receptive female (SxAT), 10 min exposure to a receptive female (CMT), or 10 min exposure to a smaller male intruder (RIT). In the control group, an observer mimicked the placement of a stimulus animal in the cage. Note that the arousal phase was omitted from the SxAT in this experiment to avoid any neuronal activation in response to consensual mating in this group. One hour after exposure to the different test conditions, animals were transcardially perfused, and brains were prepared for immunohistochemistry (see Immunohistochemistry and Microscopical analyses).

Five males were excluded from analysis due to either low overall sexual motivation during training or to suboptimal perfusion/brain tissue processing, resulting in the following group sizes: *n* = 4 (control), *n* = 12 (SxAT), *n* = 8 (CMT), *n* = 7 (RIT). The following behaviors were selected for correlation analyses: number of forced mounts (SxAT), aggressive behaviors (SxAT, RIT), and regular mounts (CMT).

#### Effects of pAIC inhibition on SxA (Experiment 5)

To test whether the pAIC—which was found to be particularly activated in response to SxA—is causally linked to the display of SxA behavior, we functionally inhibited the pAIC prior to a SxAT.

As explained above (see Experiment 3), rats bilaterally fitted with guide cannulas above the pAIC 5 days prior to training were first trained in three test sessions performed on 3 consecutive days.

To homogenize sexual behavior between subjects, we modified the training paradigm to include two CMTs (CMT-1/2) followed by one SxAT (SxAT-1). The experimental phase consisted of three consecutive daily SxAT (SxAT-2/3/4), in which rats received either a muscimol-baclofen cocktail or saline (see Infusion of baclofen and muscimol into the posterior agranular insular cortex (pAIC)) in a cross-over within-subjects design 5 min prior to either SxAT-2 or SxAT-4. SxAT-3 served as a wash-out test. Eleven rats were excluded from analyses due to either low sexual motivation during the two CMTs (*n* = 7) or misplaced guide cannula (*n* = 4). To capture the full spectrum of behaviors, all experimental SxAT’s were scored from video using J-Watcher by trained observers blind to the pharmacological treatment. The percentage of time spent displaying forced mounts, aggressive behaviors, neutral behavior and social behavior in the 10 min tests were calculated.

### Statistics

All statistics were performed using SPSS version 23 (IBM).

For experiment 1, behavioral changes over time were analyzed using a two-way mixed ANOVA with time (i.e., three consecutive daily SxAT) as within-subjects factor and surgery (none vs. i.c.v. cannula placement) as between-subjects factor. Behavior of the males in response to passive or aggressive females was analyzed using ANOVA. In addition, Pearson’s correlation analyses were performed to assess the correlations between the various behavioral outcomes.

For experiment 2, ANOVA followed by Bonferroni-corrected post-hoc pairwise comparisons were performed to assess behavioral differences in the SxAT between HAB, LAB and NAB rats. For experiment 3, ANOVA followed by Dunnett’s pairwise comparisons of treatments with VEH were performed to assess the effects of i.c.v. OXT, i.c.v. AVP, and i.p. ethanol on behavior in the SxAT. For experiment 4, ANOVA followed by Bonferroni-corrected post-hoc pairwise comparisons were performed to assess group differences in Fos-IR. In addition, Pearson’s correlation analyses were performed to assess the correlations between specific behavioral parameters and Fos-IR.

For experiment 5, within-subjects two-tailed Student’s *t* tests were used to compare behavioral scores following vehicle vs. muscimol-baclofen treatment.

## Results

### Experiment 1: Basic behavioral profile and individual differences in SxA

**Fig. 1:**
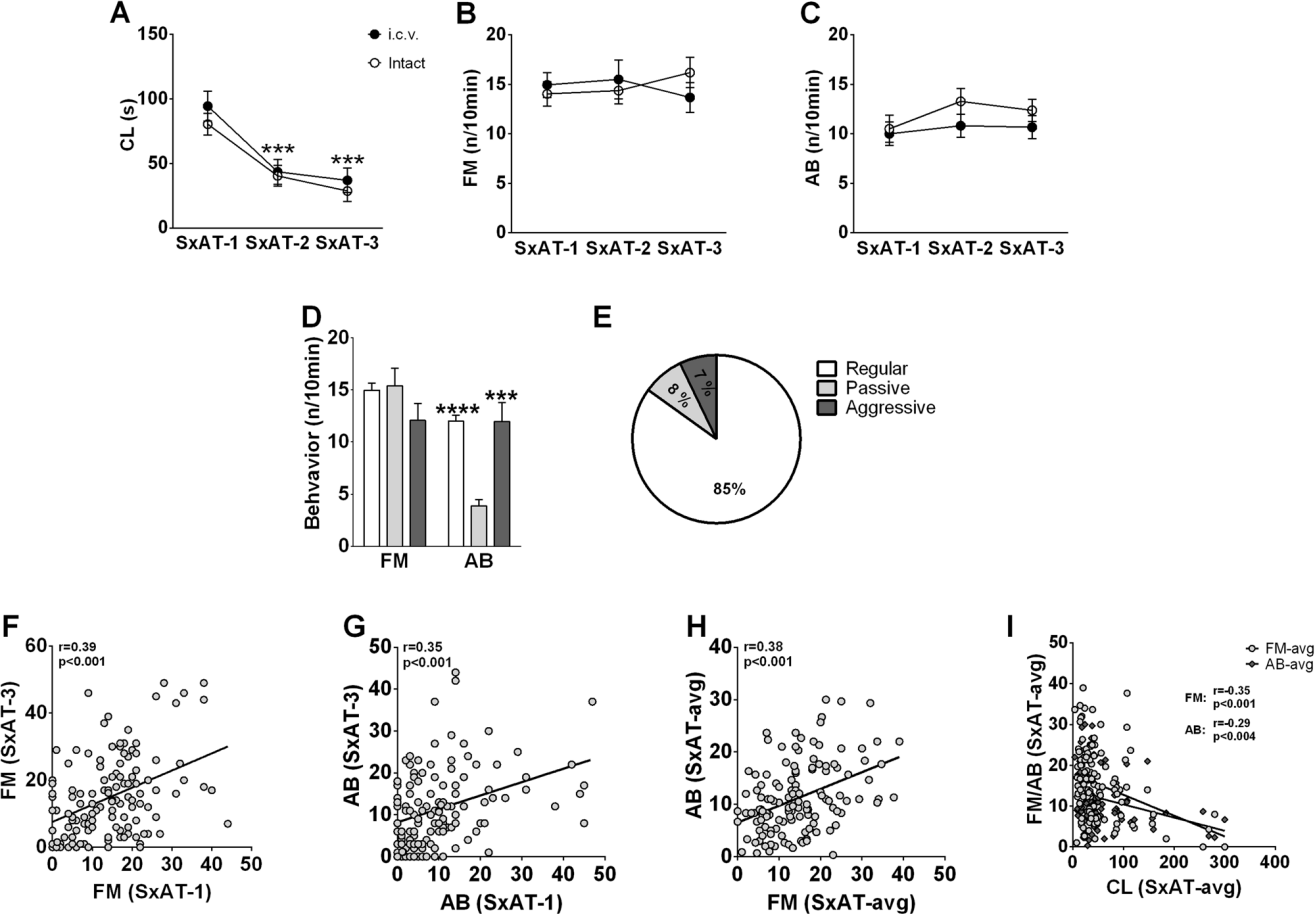
Behavioral profile of adult male Wistar rats in sexual aggression tests (SxAT). **A** Latencies to copulate (CL) with a receptive female, (**B**) frequency of forced mounts (FM), and (**C**) frequency of aggressive behavior (AB) of intact (white circles *n* = 69) and i.c.v. cannulated (black circles, *n* = 57) males toward a non-receptive female during the SxAT performed on 3 consecutive days. **D** Frequency of forced mounts (FM) and aggressive behavior (AB) of males against females with a regular, a passive, or an aggressive response type (all three SxATs combined). All data are means ± S.E.M. **E** Percentage of trials where males ecountered females displaying regular, passive or aggressive responses to the male forced mounts *n* = 378. **F** Scatter plot of FM frequencies displayed during SxAT-1 vs. SxAT-3. **G** Scatter plot of AB frequencies during SxAT-1 vs. SxAT-3. **H** Scatter plot of FM frequencies (averaged over SxAT-1, SxAT-2 and SxAT-3) vs. AB frequencies (averaged over SxAT-1, SxAT-2 and SxAT-3). **I** scatter plots of the CL vs. FM (dark-gray diamonds) or AB (gray circles) averaged over SxAT-1, SxAT-2 and SxAT-3).

The average copulation latency of the 126 male rats significantly declined over the three consecutive SxAT (F[2] = 41.909, *p* < 0.001) (Fig. 1A)). Seven males failed to start mating with the receptive female within 5 min in SxAT-1, four males failed in SxAT-2, and two males failed in SxAT-3; among them was one male, which failed in all three SxAT training sessions.There were no changes over time in the frequency of forced mounting (F[2] = 0.090, *p* = 0.914) or aggressive behavior (F[2] = 1.457, *p* = 0.235) (Fig. 1B, C, respectively). As none of the behaviors were affected by i.c.v. surgery (F[1] < 1.412, *p* > 0.246), behavioral data from intact (*n* = 69) and cannulated (*n* = 57) animals were pooled for the female response and correlation analyses.

Over all 378 SxATs performed (not separated by training day), female responses could be classified into three types (see Sexual aggression test): ‘passive’ (responding to more than 50% of forced mounts with rejection strategy 1); ‘aggressive’ (attacking the male and/or displaying 10 or more aggressive acts in an SxAT), or ‘regular’ (neither passive nor aggressive). Males encountered a passive female in 30 out of 378 SxATs (8% of the tests), and an aggressive female in 27 out of 378 SxATs (7%) (note that females never responded both passively and aggressively, and that most of the females responded regularly—85% of trials) (Fig. 1E). Males displayed the same frequency of forced mounts toward passive, aggressive, or regular females (F[2] = 0.815, *p* = 0.444; Fig. 1D), but changed their aggressive behavior dependent on the female response type (F[2] = 10.043, *p* = 0.000; Fig. 1D): passive females received less aggression from the male than either aggressive females (*p* = 0.004) or regular females (*p* < 0.001).

Relevant Pearson’s correlation coefficients among the behavioral parameters in SxAT-1-3 are summarized in Table 1 (note that the same analyses using non-parametric Spearman’s tests gave a very similar result). The forced mounting frequency scores were found to significantly correlate with each other in all three training sessions, with the strongest correlation between SxAT-1 and SxAT-3 (Fig. 1F). Also, the aggression scores correlated positively with each other in the three training sessions, with the strongest correlation between SxAT-1 and SxAT-3 (Fig. 1G). The forced mounting and aggressive behavior scores correlated positively and significantly within each training session, resulting in a significant correlation between forced mounting and aggressive behavior scores averaged over SxAT-1/2/3 (Fig. 1H). Moreover, a significant negative correlation between averaged copulation latency and forced mounting as well as aggressive behavior scores was found (Fig. 1I).

**Table 1:** Overview of Pearson’s correlation coefficients between the different behaviors (CL, FM, AB) in each of the three consecutive daily SxAT, or the average over all three SxAT.

Correlation		*r*	*p*
FM	SxAT-1 vs. SxAT-2	**0.326**	**<0.001**
	SxAT-2 vs. SxAT-3	**0.280**	**0.001**
	SxAT-1 vs. SxAT-3	**0.399**	**<0.001**
AB	SxAT-1 vs. SxAT-2	0.085	0.342
	SxAT-2 vs. SxAT-3	**0.271**	**0.002**
	SxAT-1 vs. SxAT-3	**0.351**	**<0.001**
SxAT-1	FM vs. AB	**0.241**	**0.007**
	FM vs. CL	–**296**	**0.002**
	AB vs. CL	–199	0.037
SxAT-2	FM vs. AB	**0.266**	**0.003**
	FM vs. CL	–**302**	**0.001**
	AB vs. CL	–235	0.013
SxAT-3	FM vs. AB	**0.297**	**0.001**
	FM vs. CL	–**276**	**0.004**
	AB vs. CL	–135	0.163
SxAT-avg	FM vs. AB	**386**	**<0.001**
	FM vs. CL	–**351**	**<0.001**
	AB vs. CL	–**298**	**<0.001**

Significant correlations in bold.

### Experiment 2: SxA in HAB, LAB, and NAB rats

Statistics for experiment 2 are summarized in Table 2.

**Table 2:** Overview of ANOVA and, if appropriate, post-hoc pairwise comparisons between rats selectively bred for high (HAB) vs. low (LAB) anxiety behavior or not selectively bred (NAB) of their behaviors in a 5 min EPM or 5 min SxAT.

	ANOVA		Pairwise comparisons		
Behavior	*F* (2)	*p*	HAB vs. NAB	HAB vs. LAB	LAB vs. NAB
EPM (% time in OA)	48.977	**<0.001**	**<0.001**	**<0.001**	**<0.001**
FM (SxAT-1)	0.844	0.439	–	–	–
FM (SxAT-2)	3.237	0.051	–	–	–
FM (SxAT-3)	5.480	**0.009**	0.055	**0.008**	0.750
FM (SxAT-avg)	7.611	**0.002**	**0.040**	**0.001**	0.252
AB (SxAT-1)	0.024	0.976	–	–	–
AB (SxAT-2)	5.076	**0.012**	**0.015**	1.000	0.140
AB (SxAT-3)	3.930	**0.029**	0.069	1.000	0.095
AB (SxAT-avg)	5.493	**0.008**	**0.014**	1.000	0.071
CL (SxAT-1)	9.133	**0.001**	**0.021**	0.986	**0.001**
CL (SxAT-3)	1.618	0.213	–	–	–
CL (SxAT-avg)	5.169	**0.011**	0.071	1.000	**0.020**
LTA% (SxAT-avg)	12.39	**0.001**	–	–	**<0.001**

Significant differences in bold.

HAB, LAB, and NAB rats differed in their levels of anxiety-related behavior on the EPM with HAB rats being the most anxious (% of time in open arms: 5.07 ± 1.17%), followed by NAB rats (32.35 ± 3.64%) and LAB rats (60.33 ± 3.78%).

**Fig. 2:**
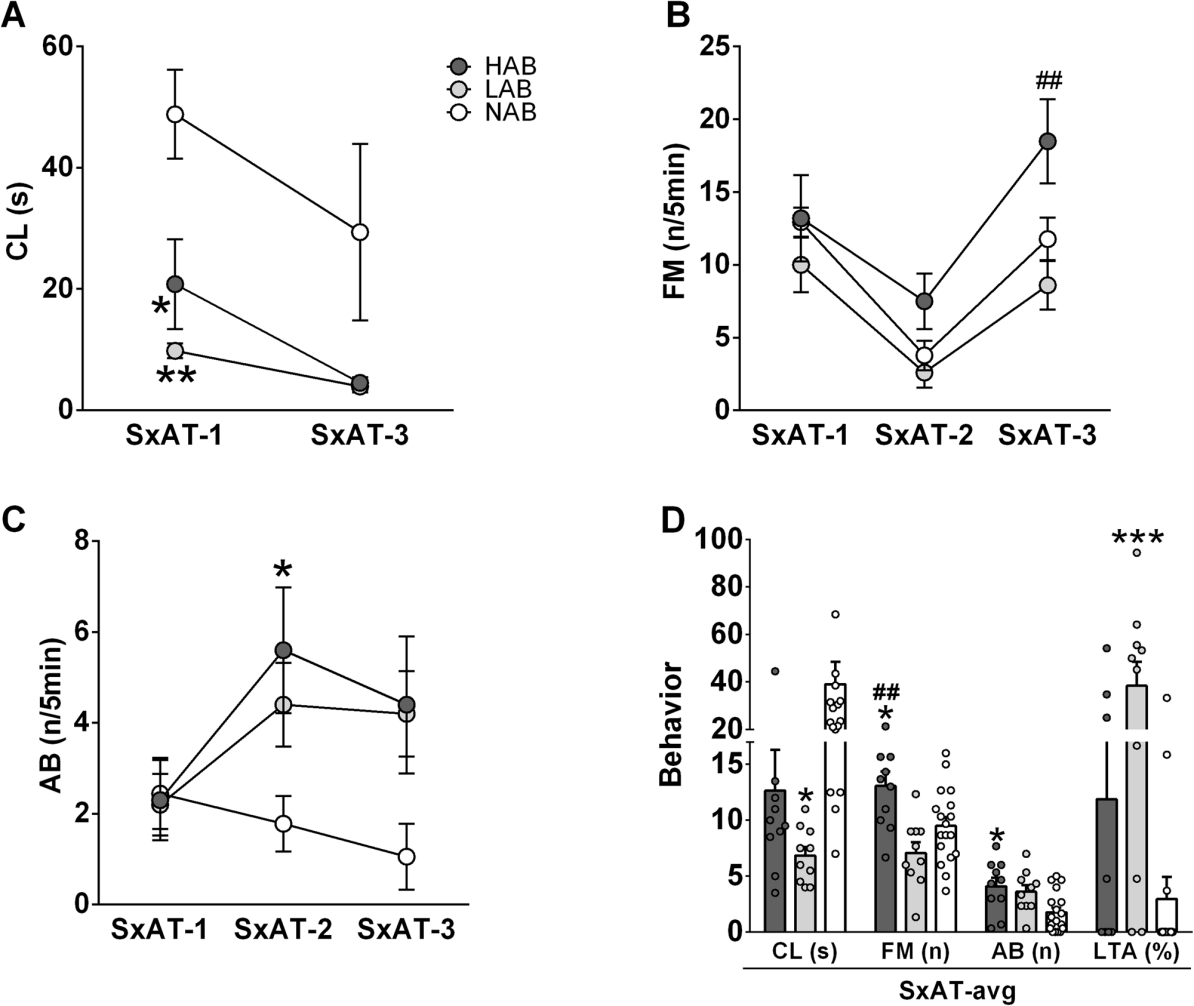
Behavioral profile of adult male HAB (dark-gray circles and bars), LAB (light-gray circles and bars) and NAB (white circles and bars) rats in three consecutive daily sexual aggression tests (SxAT-1/2/3). **A** Latency to copulate (CL) with a receptive female rat in SxAT-1 and SxAT-3 (note that SxAT-2 did not have an arousal phase). **B** Frequency of forced mounts (FM), and (**C**): Frequency of aggressive behaviors (AB) toward a non-receptive female in SxAT-1/2/3. **D** CL, FM, AB, and the proportion of lateral threats and attacks in AB (LTA%), averaged over SxAT1/2/3. Data are presented as mean ± s.e.m. *p < 0.05, **p < 0.01, ***p < 0.001 vs. NAB. ^##^*p* < 0.01 HAB vs. LAB.

In the SxAT, HAB males showed the highest forced mounting score, which differed significantly from LAB males in SxAT-3 (Fig. 2B) and from both LAB and NAB males in the average of all SxAT (Fig. 2D). HAB rats also showed the highest aggressive behavior score, which differed significantly from NAB, but not LAB, rats in SxAT-2 (Fig. 2B, note that this was the test without arousal phase) and in the average of all SxATs (Fig. 2D). NAB rats showed the longest copulation latency, which differed significantly from both HAB and LAB rats in SxAT-1 (Fig. 2A) and from LAB rats (but not HAB rats) in the average of SxAT-1 and SxAT-3 (Fig. 2D). The aggressive behavior of LAB rats had a higher proportion of lateral threats and attacks than NAB, but not HAB rats (Fig. 2D).

### Experiment 3: Pharmacological manipulation of SxA

Statistics for experiment 3 are summarized in Table 3.

**Table 3:** Overview of ANOVA and, if appropriate, post-hoc pairwise comparisons of the percentage of time displaying four main categories of behaviors (plus individual aggressive behaviors) in a 10 min SxAT in response to i.c.v. infusions of OXT or AVP vs. VEH, or IP injections of ETH vs. VEH.

	ANOVA		Pairwise comparisons	
Behavior (% of time)	*F* (2)	*p*	VEH vs. OXT-1	VEH vs. OXT-100
Forced mounting	5.054	**0.016**	**0.029**	0.893
Aggressive behavior	4.139	**0.031**	0.629	**0.019**
- threat	0.984	0.390	–	–
- keep down	2.481	0.108	–	–
- forced grooming	0.601	0.558	–	–
Neutral behavior	1.257	0.305	–	–
Social behavior	0.654	0.530	–	–
Behavior (% of time)	*F* (2)	*p*	VEH vs. AVP-0.1	VEH vs. AVP-1
Forced mounting	0.549	0.585	–	–
Aggressive behavior	0.321	0.726	–	–
- threat	0.294	0.748	–	–
- keep down	1.060	0.363	–	–
- forced grooming	1.204	0.318	–	–
Neutral behavior	0.529	0.596	–	–
Social behavior	0.824	0.451	–	–
Behavior (% of time)	*F* (2)	*p*	VEH vs. ETH-0.5	VEH vs. ETH-1.5
Forced mounting	0.169	0.845	**–**	**–**
Aggressive behavior	2.172	0.134	–	–
- threat	2.369	0.113	–	–
- keep down	1.773	0.190	–	–
- forced grooming	2.727	0.084	–	–
Neutral behavior	3.891	**0.033**	0.070	**0.030**
Social behavior	8.253	**0.002**	**0.01**	**0.001**

Significant differences in bold.

**Fig. 3:**
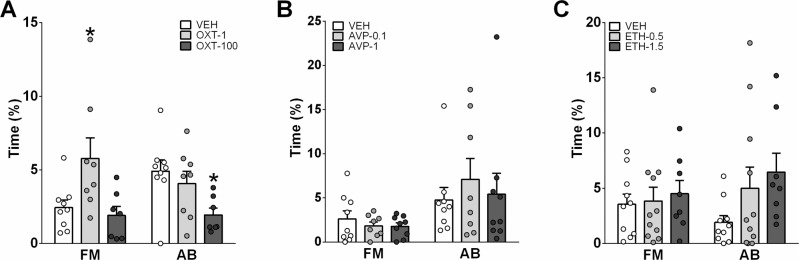
Effects of synthetic oxytocin or vasopressin, and of ethanol on sexual aggression in adult male Wistar rats. Effects of synthetic OXT and AVP, and of ethanol on sexual aggression in adult male Wistar rats. Sexual aggression was calculated as the percentage of time displaying forced mounting (FM) and aggressive behavior (AB) in a 10-min sexual aggression test (SxAT) following (**A**) i.c.v. infusion with OXT (1 or 100 ng/5 μl Ringer), **B** i.c.v. infusion with AVP (0.1 or 1 ng/5 μl Ringer), or **C** i.p. injection with ethanol (0.5 or 1.5 mg/kg ethanol), or vehicle (VEH, Ringer or saline). Data are mean + s.e.m. *p < 0.05 vs. VEH.

**OXT-treatment:** There were group differences in the percentage of time displaying forced mounting and aggressive behavior, but not in the percentage of time displaying neutral or non-aggressive social behaviors during SxAT-4 between i.c.v. OXT- and vehicle-treated rats. Specifically, OXT-1 (but not OXT-100) treatment significantly increased the percentage of time displaying forced mounting compared to vehicle, whereas OXT-100 (but not OXT-1) treatment reduced the percentage of time behaving aggressively compared to vehicle (Fig. 3A). This reduction was not caused by a specific type of aggressive behavior. Moreover, there were no group differences in consensual mating assessed in the 5 min CMT.

**AVP-treatment:** There were no group differences in any of the behavioral categories measured in the SxAT or CMT between i.c.v. AVP- and VEH-treated rats (Fig. 3B).

**Ethanol-treatment:** There were no differences in the percentage of time displaying forced mounting or aggressive behavior between ethanol- and vehicle-treated rats (Fig. 3C). However, ethanol-treatment affected the percentage of neutral and non-aggressive social behaviors: ETH-1.5, but not ETH-0.5, significantly increased neutral behaviors whereas both ETH-0.5 and ETH-1.5 reduced non-aggressive social behaviors (data not shown).

### Experiment 4: Neuronal activation in response to SxA

Statistics for experiment 4 are summarized in Table 4.

**Table 4:** Overview of ANOVA and, if appropriate, post-hoc pairwise comparisons of Fos-IR in various brain regions in response to the experimental conditions (Control, SxAT, CMT, RIT), and Pearson’s correlation coefficients between Fos-IR and individual behaviors.

Area		ANOVA		Pairwise comparisons					
		*F* (2)	*p*	CON vs. SxAT	CON vs. CMT	CON vs. RIT	SxAT vs. RIT	SxAT vs. RIT	CMT vs. RIT
LHa		0.933	0.438	–	–	–	–	–	–
CeA		0.661	0.584	–	–	–	–	–	–
MePD		1.626	0.208	–	–	–	–	–	–
PRL		5.276	**0.005**	**0.008**	**0.005**	**0.032**	1.000	1.000	1.000
pAcC		5.689	**0.004**	**0.012**	**0.006**	**0.006**	1.000	1.000	1.000
BLA		4.644	**0.010**	**0.022**	**0.008**	**0.042**	1.000	1.000	1.000
VMHvl		6.375	**0.002**	**0.004**	**0.033**	**0.002**	1.000	1.000	.981
pCg2		3.960	**0.019**	**0.014**	0.083	0.073	1.000	1.000	1.000
aAcC		4.016	**0.017**	**0.032**	0.496	1.000	1.000	0.106	1.000
aAcS		5.757	**0.004**	**0.003**	0.322	0.142	0.160	0.616	1.000
pAcS		3.258	**0.038**	**0.027**	0.230	0.234	1.000	1.000	1.000
aAIC		7.412	**0.001**	**0.003**	**0.045**	1.000	1.000	**0.013**	0.376
pAIC		6.418	**0.002**	**0.003**	0.267	0.794	0.182	**0.049**	1.000
IL		3.412	**0.032**	0.083	**0.028**	0.466	1.000	1.000	0.986
aCg1		7.687	**0.001**	0.973	**0.001**	0.602	**0.004**	1.000	**0.039**
pCg1		3.582	**0.028**	0.152	**0.027**	0.922	1.000	1.000	0.404
MPOA		3.941	**0.019**	1.000	**0.033**	1.000	0.063	1.000	0.212
LSV		6.351	**0.002**	0.218	**0.002**	0.067	**0.047**	1.000	0.564
LSD		4.701	**0.010**	0.150	0.178	**0.006**	1.000	0.353	0.619
AHA		3.737	**0.023**	0.174	0.236	**0.015**	1.000	0.799	0.894
LHb		3.844	**0.022**	0.085	0.243	**0.017**	1.000	1.000	0.919
**Area vs. Behavior**		**Pearson’s correlation coefficients**							
		**ALL**		**CMT**		**RIT**		**SxAT**	
		***r***	***p***	***r***	***p***	***r***	***p***	***r***	***p***
aAIC	(F)M	0.126	0.531	0.247	0.555	0.517	0.235	0.052	0.837
	AB	–075	0.711	–	–	–165	0.724	**0.713**	**0.009**
pAIC	(F)M	0.299	0.137	**0.738**	**0.036**	0.093	0.844	0.064	0.851
	AB	0.068	0.742	–	–	–608	0.147	**0.652**	**0.030**
LSd	(F)M	–101	0.632	**0.858**	**0.013**	–0.07	0.882	–0.077	0.823
	AB	0.291	0.159	–	–	–441	0.322	**0.719**	**0.013**
AHA	(F)M	–178	0.383	**0.781**	**0.022**	–470	0.288	–0.268	0.425
	AB	**0.518**	**0.007**	–	–	0.707	0.076	**0.687**	**0.019**

Significant differences and correlations in bold.

**Fig. 4:**
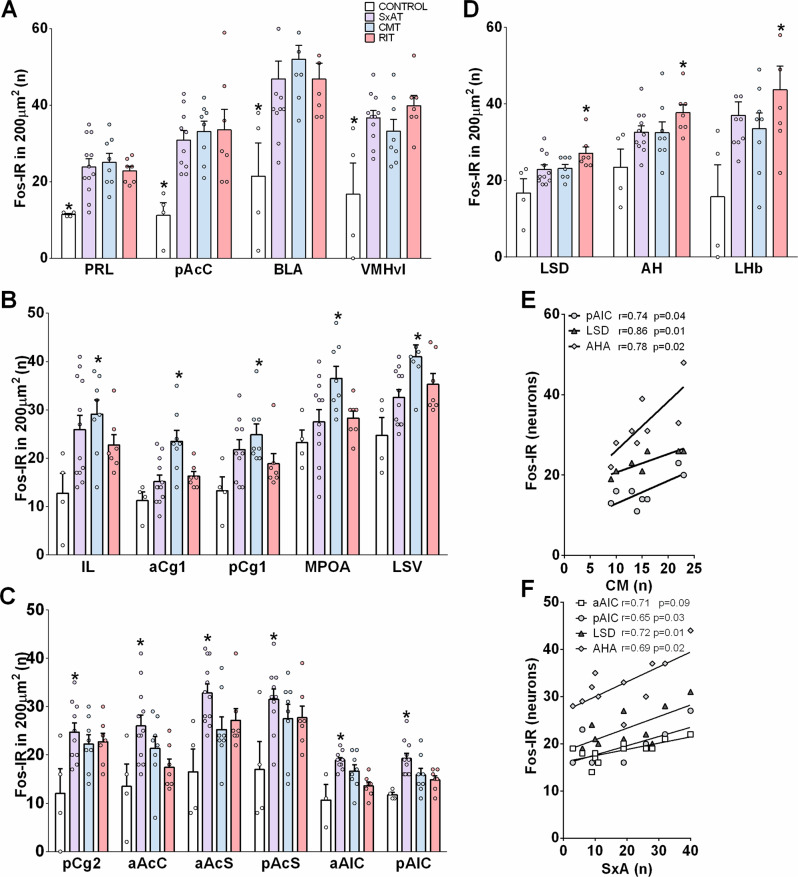
Neuronal activity in selected brain regions in response to sexual aggression. Neuronal activity was indicated by the number of Fos-IR neurons in response to 10 min of rest (CONTROL) or of exposure to the sexual aggression test (SxAT, purple bars), consensual mating (CMT, blue bars) or resident-intruder test (RIT, red bars). Brain areas are grouped according to activation patterns: **A** regions activated in all conditions, **B** regions activated exclusively by CMT, **C** regions activated only during the SxAT, and **D** regions activated only during the RIT. Data are presented as mean + s.e.m.; *p < 0.05 vs. CONTROL. Scatter plots of the number of consensual mounts (CM) in the CMT (**E**), or of aggressive behavior (**F**) in the SxAT vs. the number of Fos-IR in the aAIC, pAIC, LSD, and AHA. PRL pre-limbic cortex, IL infra-limbic cortex, aCg1 anterior cingulate cortex 1, pCg1 and pCg2 posterior cingulate cortex 1 and 2, respectively, aAIC and pAIC anterior and posterior agranular insular cortex, respectively, aAcC and pAcC anterior and posterior nucleus accumbens core, respectively, aAcS and pAcS anterior and posterior nucleus accumbens shell, BLA basolateral amygdala, CeA central amygdala, MeApd posterodorsal medial amygdala, LSD dorsal lateral septum, LSV ventral lateral septum, AH anterior hypothalamic attack area, LHA lateral hypothalamic attack area, LHb lateral habenula, MPOA medial preoptic area, VMHvl ventrolateral ventromedial hypothalamus.

ANOVA analysis revealed group differences in Fos-IR in almost all of the analyzed areas, except for the LHA, CeA, and MePD. In areas with group differences, four general statistical patterns could be observed (Fig. 4). Pattern 1 was a similar increase in Fos-IR in response to all behavioral tests compared to control conditions, which was seen in the PRL, pAcC, BLA and VMHvl (Fig. 4A). Pattern 2 was an increase in Fos-IR specifically in response to the CMT compared to control conditions, which was seen in the IL, aCg1, pCg1, MPOA and LSV (Fig. 4B). In the aAIC, Fos-IR was also slightly increased in response to the CMT compared to control conditions. Pattern 3 was an increase in Fos-IR specifically in response to the SxAT compared to control conditions, which was seen in the pCg2, aAcC, aAcS, pAcS, aAIC and pAIC (Fig. 4C). Pattern 4 was an increase in Fos-IR specifically in response to the RIT compared to control conditions, which was seen in the LSD, AHA and LHb (Fig. 4D).

Rats displayed a considerable behavioral individual variability in the various tests, allowing the analysis of correlations between certain behaviors and Fos-IR. We chose consensual mounts (in the CMT), forced mounts (in the SxAT and RIT), sexual aggression (in the SxAT) and territorial aggression (in the RIT) as the most relevant behaviors. Pearson’s correlation analyses revealed that mounts (in all behavioral tests combined) or forced mounting (in either the SxAT or RIT) did not correlate with Fos-IR in any of the analyzed brain areas, whereas consensual mounting (in the CMT) correlated positively with Fos-IR in the pAIC, LSD and AHA (Fig. 4E). Pearson’s correlation analyses furthermore revealed that aggression (in all behavioral tests combined) correlated positively with Fos-IR in the AHA, whereas specific territorial aggression (RIT) showed no correlations with Fos-IR in any of the analyzed brain areas. Importantly, overall SxA (SxAT) correlated positively with Fos-IR in the aAIC, pAIC, LSD and AHA (Fig. 4F).

### Experiment 5: Effects of pAIC-inhibition on SxA

Statistics for experiment 5 are summarized in Table 5.

**Table 5:** Overview of *t*-test comparisons of the percentage of time displaying four main categories of behaviors (plus individual aggressive behaviors) in a 10 min SxAT in response to injection of either vehicle or a cocktail of muscimol and baclofen into the pAIC.

Behavior (% of time)	VEH	Musc/Bacl	*p*
Forced mounting	3.9 ± 0.9	4.4 ± 1	0.628
- forced grooming	0.9 ± 0.6	0.3 ± 0.1	0.37
Aggressive behavior	6.5 ± 1.3	5.7 ± 3.7	0.745
- threat	3.3 ± 0.5	1.7 ± 0.4	**0.039**
- keep down	1.9 ± 0.6	2.7 ± 0.1	0.99
Neutral behavior	65.9 ± 1.4	65.01 ± 3.4	0.765
Social behavior	17 ± 2.9	13.1 ± 2.1	0.291

Significant differences in bold.

**Fig. 5:**
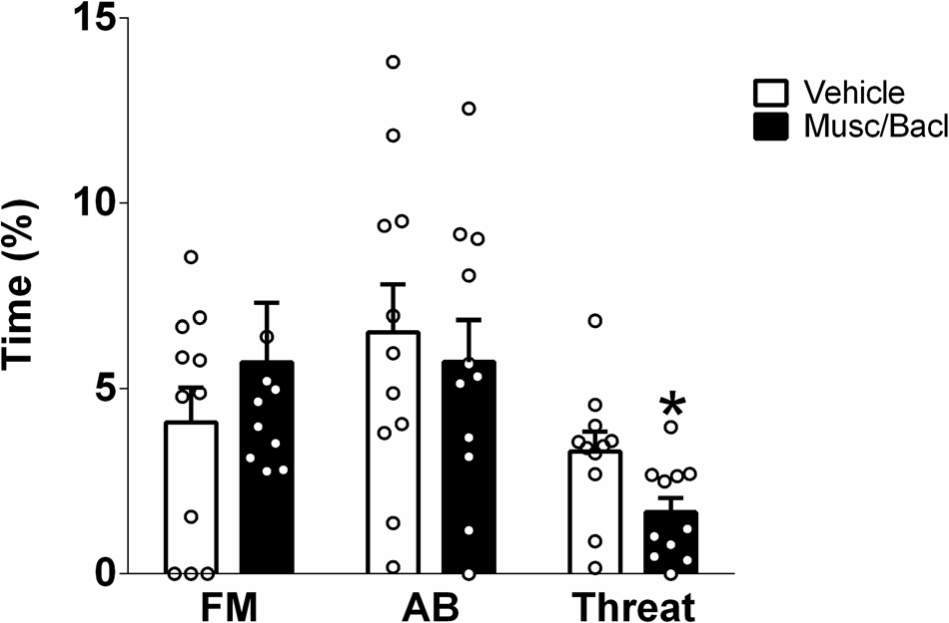
Effects of pharmacological inhibition of the posterior agranular insular cortex (pAIC) on sexual aggression (SxA) indicated by the percentage time displaying forced mounting (FM), and on general aggressive behavior (AB) and threat. Adult male Wistar rats were bilaterally infused with a cocktail of 20 ng/μl muscimol and 200 ng/μl baclofen or vehicle into the pAIC 10 prior to exposure to the SxAT. Data are presented as mean + s.e.m.; *p < 0.05.

Inhibition of pAIC did not change the percentage of time displaying forced mounting or aggressive behavior in the SxAT, but it significantly decreased one type of aggression behavior: threats toward the female (two-tailed Student’s *t*-test t_(10)_=2.36, *p* = 0.039; Fig. 5),

## Discussion

The experimental results reported in this paper indicate that a simple confrontational behavioral paradigm, i.e., the sexual aggression test (SxAT) in rats can be used as a promising and reliable animal model to study basic aspects of SxA and its neurobiological correlates. This is of high translational relevance, as animal models have proven to be essential instruments in the understanding of the mechanisms and biological background of virtually all human behaviors, with the notable exception of sexual violence. Our results show that SxA, i.e., a combination of forced mounting and aggressive behavior toward a non-willing female, are readily displayed by sexually aroused male Wistar rats and can be reliably quantified in the SxAT. Although forced mounting and aggressive behavior varied considerably between individual rats, and from one cohort to another, we found overall tendencies when analyzing a large number of rats or comparing rats with inbred extremes in anxiety and aggressive behaviors, i.e. HAB and LAB rats. Moreover, SxA displayed during the SxAT was found to be stable enough to distinguish the effects of pharmacological manipulations. We could further identify specific neuronal activation patterns related to SxA, which could be clearly distinguished from those related to territorial aggression and consensual mating, indicating that the pAIC and the nucleus accumbens are most likely to be implicated. Indeed, inhibition of the pAIC decreased threat behavior in the SxAT, however, other parameters of SxA remained unchanged.

Experiment 1 revealed that SxA is a behavior spontaneously displayed by most naive male rats without a learning period, which remains stable over three consecutive SxAT. Thus, rats displaying a high spontaneous propensity for persistent forced mounting tended to keep this behavior over time, and also showed a higher propensity for aggressive behavior toward the female, suggesting either a common cause for both behaviors or one behavior instigating the other. Furthermore, forced mounting was negatively correlated with the latency to copulate with a receptive female in the arousal phase. Combined, these correlations indicate that sexually motivated rats (defined by a low average copulation latency) show an increased tendency to persist in mounting a non-willing female, resulting in repeated rejection that likely elicits the elevated level of aggression. Conversely, rats with lower sexual motivation (defined by a high average copulation latency) tend to show lower levels of both forced mounting and aggressive behavior. Notably, not all males followed this behavioral pattern: some males that were slow to mount receptive females were much more eager to mount non-receptive females, other males displayed above-average aggressive behavior combined with below-average forced mounting or vice versa. This may be caused by the natural tendencies of the male, but it could also be (partly) caused by the variability in cues and behaviors of the stimulus females. Thus, females responding very passively to the forced mounting by the male were less likely to elicit an aggressive response. In addition, some of the stimulus females may have been physically in estrus, despite behaving non-receptively, providing additional stimulation for the male to keep mating. To reduce variability in the SxAT, it is advised to remove very passive stimulus females from an experiment, and to perform vaginal swabs (in combination with the receptivity screen tests) to remove non-receptive females that are physically in estrus.

In experiment 2, the conclusions from the first experiment could be confirmed. The increased sexual motivation (i.e., reduced copulation latency) of both HAB and LAB males was accompanied by increased SxA compared with NAB rats. The association between low copulation latency and high forced mounting and—to a lesser extent—high aggressive behavior was especially evident in HAB rats, whereas LAB rats showed a less typical pattern consisting of high sexual motivation, average forced mounting, and increased aggression with a high proportion lateral threats and attacks toward the females. These results correspond with earlier findings of increased territorial aggression in male HAB and LAB rats, and abnormal aggression (including the attack of female intruders) displayed by LAB rats in the RIT [21, 27]. The relevance of these results in selectively bred rats is that the increased SxA appears to tie in with the other abnormal social and emotional behaviors of HAB and LAB rats. The precise role of trait anxiety in the propensity for SxA, which has been found in humans as well [51], deserves more attention in future research.

Experiment 3 was designed as a first approach to quantify the potential facilitation or inhibition of SxA in the SxAT induced by pharmacological manipulations. Based on their proven capacity to promote sexual behavior (OXT [19, 24]) and to differentially modulate territorial aggression (OXT and AVP [19, 25, 26, 30–33, 52]), and other relevant social behaviors [18, 19, 53, 54], the OXT and AVP systems were first selected as targets. Acute i.c.v. infusion of synthetic AVP did not alter any aspect of SxA in the SxAT. Acute i.c.v. infusion of a low (but not high) dose of OXT, on the other hand, facilitated forced mounting, while inhibiting aggressive behavior, weakening the correlation between these behaviors seen in non-treated rats in Exp. 1. However, the reduction in aggression could not be pinned down to a specific type of aggressive behavior. Although these results suggest that OXT neurotransmission promotes the sexual aspects of SxA, while inhibiting the aggressive aspects, it is only a very first step toward unraveling the precise mechanisms, since behavioral effects of OXT are well known to be brain region-, housing and context-dependent [55–57] [22, 26, 58].

We also tested the effects of ethanol on SxA, because of its involvement in rape and assault cases in humans [29] as well as its known (dis-)inhibitory effects on aggression and sexual behavior in rodents [34–36]. We found that ethanol, in the chosen dose and treatment regimen, did neither facilitate nor inhibit forced mounting or aggressive behavior in the SxAT, although it did increase the variability in aggressive behavior. The lack of a “simple” effect of ethanol on SxA in the SxAT does not preclude the translational value of the test, as its role in human sexual assault and rape is also complex and influenced by multiple factors such as intoxication of the victims and personality traits and/or early life experiences of the perpetrator [29]. More sophisticated experiments might be able to pinpoint the effects of ethanol on SxA. For example, it may be necessary to pre-select experimental males with a similar response to ethanol, to reduce the strong individual differences that have been shown in rodents [34, 35]. It may also help to induce a stable level of inhibition experienced by experimental males in the agonistic phase, for example by implementing a learning protocol [36].

We do encourage other groups to implement the SxAT in pharmacology-based behavioral experiments, similar to tests for sexual behavior or territorial aggression. Aside from the recommendations regarding the selection of stimulus females and experimental males described above, we also want to advise the following in order to reduce individual variability which may obscure pharmacological effects: (i) increased initial group sizes will enable the selection of individuals with reliable SxA displayed during the training phase; (ii) prolonged social isolation prior to the training may stimulate robust SxA [26, 31]; (iii) extensive post-surgical habituation is likely to reduce stress-induced inhibition of SxA.

Experiment 4 revealed that exposure to the SxAT in general, and the display of SxA in particular, induce a specific neuronal activation pattern that only partly overlaps with that induced by consensual mating (CMT) or territorial aggression (RIT). The results implicate a specific role of the cingulate cortex, the nucleus accumbens core and shell, and most prominently the agranular insular cortex in SxA. The insular cortex is the core brain area involved in social decision-making, i.e., the facilitation or inhibition of behaviors by positive or negative cues from conspecifics [43, 59]. Indeed, exposure to the SxAT can be viewed as a prime example of social decision-making: the clear negative cues from the non-receptive female (kicking, moving the genitals away from the male, audible distress vocalizations) inhibit the ongoing sexual behavior of the male with various degrees of effectiveness depending on (presumably) the male’s sexual motivation and empathic sensitivity toward negative cues, combined with the strength of the cues from the female.

Despite the promising pattern of neuronal activity in the pAIC, we only found very mild effects of the inhibition of the pAIC on SxA, i.e., a reduction in threat behavior. We consider the present experiment as a very first step into the unraveling of the role of the pAIC and associated areas in SxA. Thus, the pAIC may be predominantly be activated by (forced) mounts (as the neuronal activation also correlated with consensual mounting), without playing a decisive role in the execution of the behavior. Alternatively, local drug infusion will target all neuronal populations of the pAIC, which is known to be a heterogeneous brain region with different layers and presumably various neuronal populations [42, 60], resulting in a combination of inhibition and disinhibition. More sophisticated experimental approaches are needed to determine if the activation or inhibition of selective pAIC neurons is able to change the direction of behavior in the SxAT.

Taken together, the present experiments introduce the SxAT as a viable protocol for studying SxA. It uses a behavior readily displayed by male rats and can be easily reproduced by any lab experienced in rat behavioral analyses. We acknowledge that the conflict situation between sexually aroused males and non-receptive females is artificially enforced in the SxAT, but the ensuing SxA is constistent and stable and can be utilized to learn more about the biological mechanisms that may facilitate or inhibit this behavior. In addition, the SxAT may serve a more general purpose to understand (anti-)social decision-making, as the test requires one individual to display actions that harm another individual, despite the clear distressing responses of the victim (with the observed loud distress vocalizations as a particularly interesting parameter). Furthermore, by shifting focus onto the females and looking at the acute and long-term effects of being exposed to the SxAT, this paradigm could also be a relevant addition to study the behavioral, neurobiological, and hormonal consequences of sexual [11], as compared to non-sexual, defeat [61–64].

Thus, the SxAT described and established here is the first attempt to model and gain knowledge of human sexual violence in a translational manner. We do not expect it to completely cover all (patho-)psychological aspects of human rape and sexual assault. Rather, the SxAT should be considered as a novel, useful and manageable tool to help fill the current gap in the toolbox of behavioral neuroscientists and neuroendocrinologists. By implementing it in many different paradigms and contexts, and adjusting it if necessary to cover specific aspects of SxA, we hope that it will provide some valuable answers that cannot be obtained otherwise.

## Supplementary information

Supplementary information

Supplementary video 1
