# Correction: Modelling sexual violence in male rats: the sexual aggression test (SxAT)

**DOI:** 10.1038/s41398-022-01980-4

**Published:** 2022-05-30

**Authors:** Vinícius E. de M. Oliveira, Trynke R. de Jong, Inga D. Neumann

**Affiliations:** 1 Department of Behavioural and Molecular Neurobiology, University of Regensburg, 93040 Regensburg, Germany; 2 Laboratory of Neuroendocrinology, GIGA-Neurosciences, University of Liege, 4020 Liege, Belgium; 3 Medische Biobank Noord-Nederland B.V, 9713 Groningen, Netherlands

**Keywords:** Neuroscience, Pharmacology, Psychiatric disorders

Correction to: *Translational Psychiatry* 10.1038/s41398-022-01973-3, published online 18 May 2022

The original version of this article contained an error in an author name. The correct full author name is Vinicius Elias de Moura Oliveira. Thus the name in the manuscript should be Vinícius E. de M. Oliveira. The original article has been corrected.

